# Factors Associated with Seasonal Food Insecurity among Small-Scale Subsistence Farming Households in Rural Honduras

**DOI:** 10.3390/ijerph17030706

**Published:** 2020-01-22

**Authors:** Warren Dodd, Marvin Gómez Cerna, Paola Orellena, Sally Humphries, Margaux L. Sadoine, David Zombré, Kate Zinszer, Amy Kipp, Donald C. Cole

**Affiliations:** 1 School of Public Health and Health Systems, University of Waterloo, Waterloo, ON N2L 3G1, Canada; 2 La Fundación para la Investigación Participativa con Agricultores de Honduras (FIPAH), La Ceiba M5S 2E8, Honduras; 3 Department of Sociology and Anthropology, University of Guelph, Guelph, ON N1G 2W1, Canada; 4 Department of Social and Preventive Medicine, University of Montreal, Montréal, QC H3C 3J7, Canada; 5 International Program Evaluation Unit, Centre for Global Child Health, The Hospital for Sick Children, Toronto, ON M5G 2L3, Canada; 6 Dalla Lana School of Public Health, University of Toronto, Toronto, ON M5T 3M7, Canada

**Keywords:** food access, food availability, food stability, small-scale subsistence agriculture, rural livelihoods, poverty, seasonality, migration, Central America

## Abstract

In the context of climate change, a nutritional transition, and increased pressures to migrate internally and internationally, this study examined the relationship between seasonal food insecurity and demographic, socioeconomic, and agricultural production factors among small-scale subsistence farmers in rural northern Honduras. Anchored by a partnership with the Fundación para la Investigación Participativa con Agricultores de Honduras (FIPAH) and the Yorito Municipal Health Centre, a cross-sectional household survey was administered in Yorito, Honduras, in July 2014. The study population included 1263 individuals from 248 households across 22 rural communities. A multivariate mixed effects negative binomial regression model was built to investigate the relationship between the self-reported number of months without food availability and access from subsistence agriculture in the previous year (August 2013–July 2014) and demographic, socioeconomic, and agricultural production variables. This study found a lengthier ‘lean season’ among surveyed household than previously documented in Honduras. Overall, 62.2% (95% confidence interval (CI): [59.52, 64.87]) of individuals experienced at least four months of insufficient food in the previous year. Individuals from poorer and larger households were more likely to experience insufficient food compared to individuals from wealthier and smaller households. Additionally, individuals from households that produced both maize and beans were less likely to have insufficient food compared to individuals from households that did not grow these staple crops (prevalence ratio (PR) = 0.83; 95% CI: [0.69, 0.99]). Receiving remittances from a migrant family member did not significantly reduce the prevalence of having insufficient food. As unpredictable crop yields linked to climate change and extreme weather events are projected to negatively influence the food security and nutrition outcomes of rural populations, it is important to understand how demographic, socioeconomic, and agricultural production factors may modify the ability of individuals and households engaged in small-scale subsistence agriculture to respond to adverse shocks.

## 1. Introduction

Food availability and access are interrelated in subsistence agricultural systems, as subsistence farmers are the producers and consumers of their main sources of food. To maintain household food security, subsistence farmers globally are faced with the ongoing challenge of balancing crop production for both sustenance and income [1]. The ability to utilize food produced through subsistence agriculture in a nutritionally and culturally significant way is another critical dimension of food security for households reliant on small-scale subsistence agriculture, with food security defined not only as having an available and accessible food supply, but one that is culturally preferred and nutritious [1,2]. For subsistence farmers, this can mean producing crops that are culturally significant, in spite of economic disincentives to do so [3,4]. This multidimensional understanding of food security within subsistence agricultural systems aligns with the Food and Agriculture Organization’s definition of food security, which states that “food security exists when all people, at all times, have physical and economic access to sufficient, safe and nutritious food that meets their dietary needs and food preferences for an active and health life [5].” 

In the context of a changing climate, the stability of food availability, access, and utilization over time is an aspect of food security that is increasingly challenged in subsistence agricultural systems [1,6,7]. Across Central America, the ‘lean’ or ‘hungry’ season has been documented as a time in which food availability and access among many households reliant on subsistence agriculture is insufficient [1,6,8,9]. Local food prices often rise during the ‘lean’ season, exacerbating experiences of food insecurity among small-scale subsistence farming households with limited agricultural production and storage capacity [6]. 

In Honduras, approximately 45% of the country’s nine million residents live in rural areas [10], with the majority of rural households relying on small-scale subsistence agriculture for food security [11]. For these farmers, seasonal food insecurity is commonly experienced during *los junios*, an annual period beginning every June during when the previous harvest is used up and the subsequent harvest has not yet begun [6,12]. Despite efforts to diversify rural livelihoods through non-agricultural employment (e.g., jobs in forestry or day labour opportunities), reliance on remittances from migrating family members, and small business opportunities (e.g., shop-keeping, participation in tourism), many challenges remain that contribute to persistent poverty and food insecurity in rural areas of Honduras [11,13,14]. Rural regions are often geographically remote, with the majority of infrastructure and transportation systems in Honduras concentrated in the north and the centre of the country [11,15]; this remoteness limits access to markets and economic opportunities, as well as basic services such as healthcare and education [13,15]. Access and ownership of fertile, arable land is often limited, and rural households typically have small, marginal land areas in environmentally fragile locations [13,16,17]. 

These challenges in rural regions of Honduras have been exacerbated by a number of factors, including: structural adjustment policies that have reduced state involvement in rural development [18,19]; continual price fluctuations of many foods, goods, and agricultural commodities; and, increased competition from regionalized and globalized agricultural markets [1,17]. One strategy for navigating the impacts of these economic pressures, including increased food insecurity, has been for individuals from rural households to migrate both internally and internationally in search of enhanced socioeconomic opportunities [20,21]. This outmigration has led to several social and economic transitions in rural regions, including the introduction of remittances to rural economies, shortages in manual labour for subsistence agricultural work, and changing inter- and intra-household dynamics [20,22,23,24]. For example, migration may enhance the vulnerability of those ‘left behind’ in rural communities, as households with a migrating family member may be faced with increased workloads, short-term financial and asset strain, food insecurity, and increased anxiety [1,20,22,23,24]. 

Amidst rural transitions, changing climatic conditions (such as shifting precipitation patterns, rising temperatures, and the increased prevalence and severity of extreme weather conditions) can result in substantial risks for small-scale subsistence farmers [13,16,25]. Climatic changes have caused hazardous events including droughts, hurricanes, floods and landslides, which have been associated with decreased stability and predictability of agricultural seasons [13,26]. Subsistence agricultural systems are especially vulnerable to these changes and events, owing to their frequent reliance on natural resources and ecosystem services, dependence on rain-fed crops, location on marginal lands, and limited adaptation capacity [6,17]. Of particular concern for small-scale subsistence farmers in rural Honduras is the reoccurring and intensifying drought-like conditions linked to El Niño climatic oscillations [1,9,27]. Previously, such climatic conditions have devastated crop production and worsened the effects of the ‘lean’ season. Maize and beans—both staples in the diets and cultural practices of rural Hondurans and the main crops produced by subsistence farmers—are specifically sensitive to rising temperatures and reduced precipitation [28]. Thus, climate change and the related socioeconomic factors and processes in rural Honduras are closely connected to food insecurity among households reliant on small-scale subsistence agricultural systems, influencing food availability, access, utilization, and stability [1].

For individuals and households involved in small-scale subsistence agriculture in rural Honduras, food security is defined as having ongoing, stable food availability and access, in addition to consuming culturally preferred food sources produced through subsistence agriculture. This study builds from and moves beyond exclusively production-oriented understandings of subsistence agricultural systems to quantitatively model seasonal food availability and access among individuals from households involved in small-scale subsistence agriculture. Additionally, we consider the cultural significance of specific subsistence food crops in the interpretation of our quantitative model. In bringing these components together, we are able to generate a multidimensional understanding of seasonal food insecurity in this context, which considers the interplay of climatic, cultural, demographic, socioeconomic, and agricultural production factors for small-scale subsistence agriculture. 

## 2. Materials and Methods

### 2.1. Study Partnership and Design

This study was designed in partnership with the Fundación para la Investigación Participativa con Agricultores de Honduras (FIPAH; the Foundation for Participatory Research with Honduran Farmers) and the Yorito municipal health centre. FIPAH is a Honduran non-governmental organization that has facilitated agricultural and community development activities in Yorito and in other rural areas across Honduras over the past 25 years. The analysis was based on data collected from a representative cross-sectional household survey, conducted in July 2014. Interpretation of data is supported by observations made by FIPAH staff through their ongoing agricultural and community development efforts and engagement in the region where this study was conducted.

### 2.2. Study Location

This study was conducted in 22 remote communities in the rural municipality of Yorito, Yoro, Honduras. Physical and social infrastructure in these communities is characterized by poor road access, partial electrification, and limited social and health services. According to the 2013 municipal census, there was a population of 16,482 individuals and 3956 households in Yorito. Among these households, 71.8% experienced economic hardship, 39.0% had poor quality housing, 24.0% experienced overcrowding, 17% did not have access to a latrine, and 10.9% did not have access to a stable source of water for household use.

### 2.3. Sampling and Data Collection

Multi-stage random sampling was used to select communities, and then households within communities for inclusion in the study. First, approximately half of the communities in the municipality of Yorito were randomly selected from the full list of communities provided by the 2013 census. Approximately 10% of households within each selected village were then systematically randomly sampled. When available, maps of each community were used to pre-select every tenth house based on 2013 census data. The female or male household head was asked to respond to the survey and acted as the proxy respondent for all household members. 

A previously used household survey exploring different dimensions of rural livelihoods [29,30] was adapted for the specific context of Yorito with input from FIPAH staff and the Yorito municipal health centre. The survey explored several broad domains including: demographic information, socioeconomic status, migration dynamics, livelihood diversification, self-rated health, food security, dietary diversity, and access to public services. The survey was piloted with four households and further refined following the pilot phase. Household surveys were conducted by trained local public health promoters from the Yorito municipal health centre. There were no refusals to participate in the study (i.e., 100% response).

### 2.4. Statistical Analysis

#### 2.4.1. Outcome 

The main outcome was the self-reported number of months without sufficient food availability and access for the household from the household’s subsistence agriculture production, between August 2013–July 2014 [14]. Respondents were asked, “In the last 12 months, which months did your household not have sufficient food from your own agricultural production?” Values ranged from zero (i.e., no months without sufficient food availability and access) to 12 (i.e., full year without sufficient food availability and access). Specific months (e.g., June, July, etc.) without sufficient food availability and access for the household were also provided.

#### 2.4.2. Demographic, Socioeconomic, and Agricultural Production Predictors

Recognizing the multidimensional nature of seasonal food availability and access [1,6,14], in addition to our experiences with agricultural development in this context, we selected variables useful for predicting seasonal food availability and access in rural Honduras in ways relevant for the analysis. These predictors included demographic (e.g., sex, literacy, marital status, number of household members), socioeconomic (e.g., wealth index, land ownership, sources of household income, presence of migrant member in the household), and agricultural production variables (e.g., amount of land under cultivation, crop type). Amount of land under cultivation was measured in manzanas (0.7 of a hectare).

#### 2.4.3. Analysis

We conducted principal component analysis to construct a household wealth index [31]. The components of the household wealth index included self-reported data on house and land ownership, household assets which were recoded as binary variables (e.g., bicycle, motorcycle, refrigerator, television, mobile phone), possession of livestock (e.g., poultry, pigs, cows, horses), quality of housing (e.g., roof, floor, and wall materials), access to drinking water on the household’s property, and access to water sanitation services. The principal component analysis was applied to 16 asset indicator variables that showed a relevant contribution to the combined wealth index. The Kaiser–Meyer–Olkin (KMO) test of the sampling adequacy was medium (0.69) and the Bartlett test of sphericity was significant (*p* < 0.001), both indicating the adequacy of the data for factor analysis [32]. The first principal component (explaining 29% of the variation in the data set) with the highest eigenvalue (4.64) was categorized into quintiles and used as a proxy indicator for the household socioeconomic status [31].

A negative binomial regression was selected over Poisson regression because the unconditional mean of the outcome variable was lower than its variance [33]. A bivariate analysis was conducted to examine unadjusted relationships between the number of months without sufficient food from subsistence agriculture and relevant demographic, socioeconomic, and agricultural production variables. Then, a multivariable analysis was performed with all the selected variables. Using a backward stepwise process, factors found to be associated with the outcome of interest at *p* < 0.1 were retained in the multivariable models. Several variables related to land ownership and crop cultivation were strongly correlated. Thus, we tested variables for collinearity at the r > 0.5 level, and for multicollinearity by examining the variance inflation factor [33]. To account for collinearity among variables related to land ownership and cultivation, we excluded these variables from the final model. To account for collinearity among different types of crops grown by households, we created a categorical variable that allowed us to differentiate between the relative contribution of maize and beans to seasonal food availability and access. Additionally, this approach allowed us to consider how the diversification of staple crops may influence seasonal food availability and access. 

Given the hierarchical nature of the data (as a result of the sampling process whereby individuals were nested within households, which were nested within communities), individuals from the same household or community were more likely to be similar to each other than individuals from different households or communities. Ignoring these clustering effects could lead to the underestimation of standard errors and the assumption of statistical significance where it did not exist [33]. The multilevel, mixed effects model enabled us to control for clustering effects of households and communities and correct for standard errors in these levels in order to obtain coefficients with more accuracy [34]. 

The exponentiated coefficients arising from the multivariate mixed effects negative binominal regression model were interpreted as adjusted prevalence ratios (PR) with corresponding 95% confidence intervals (CIs). The goodness-of-fit of this model was tested using the Bayesian information criteria with the most parsimonious model presented.

All statistical analyses were performed using version 15.0 of the Stata/MP software package (StataCorp, College Station, USA).

### 2.5. Ethical Statement

This project was reviewed and approved by the Research Ethics Board at the University of Guelph, Canada. Prior to commencing a household survey, informed oral consent was obtained.

## 3. Results

### 3.1. Demographic Information

In total, 1263 individuals from 248 households residing in 22 rural communities in the municipality of Yorito were included in the study. Households had an average of 5.1 members (± 1.94). Primary sources of income for households included agriculture (61.3%) and local agricultural day labour (*jornalero*; 58.3%). Additionally, 47 households (19.0%) had a least one migrant member at the time of survey administration. The majority of migration was internal to nearby urban centres in Honduras.

### 3.2. Seasonality of Food Availability and Access

**Figure 1:**
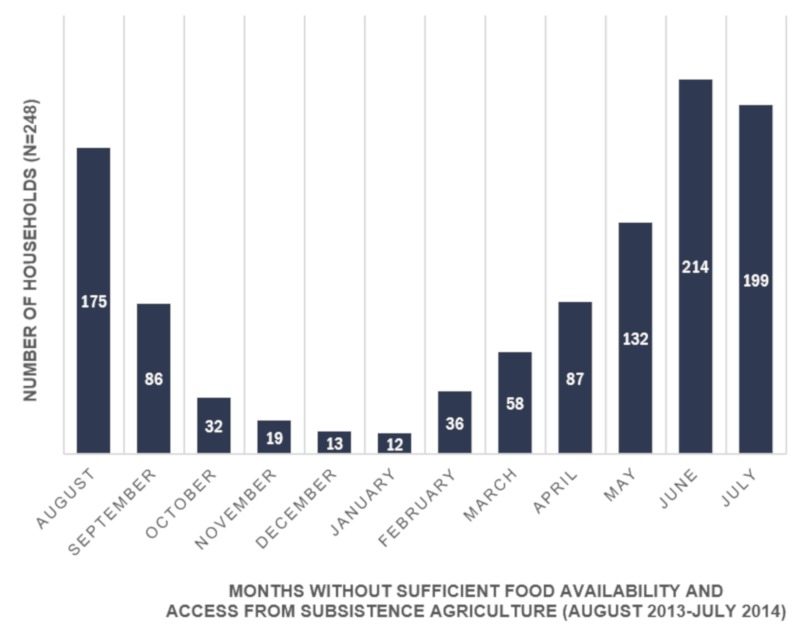
Seasonality of food availability and access from subsistence agriculture in Yorito, Honduras, August 2013–July 2014 (n = 248 households).

Food availability and access from subsistence agriculture was seasonal in nature. Food availability and access was highest in January and lowest in June with 214 households (86.3%) reporting insufficient food during this month (see Figure 1). On average, households experienced 4.3 months (± 2.42) with insufficient food during the period of August 2013–July 2014. At the individual level, only 4.4% of individuals had sufficient food availability and access from subsistence agriculture for the entire year (95% CI: [3.43, 5.72]). Conversely, 33.3% of individuals experienced 1 to 3 months without sufficient food (95% CI: [30.78, 35.99]), and 62.2% of individuals experienced over 4 months without sufficient food (95% CI: [59.52, 64.87]) in the previous year.

### 3.3. Factors Associated with Seasonal Food Availability and Access

Table 1 shows the bivariate associations between the number of months with insufficient food availability and access from subsistence agriculture in the previous year and demographic and socioeconomic predictors. Individuals who experienced four months or more of insufficient food from subsistence agriculture were commonly from the largest and poorest households. Indeed, 70.2% (95% CI: [65.3, 74.6]) of individuals from households with six to seven members, and 72.6% (95% CI: [66.4, 78.0]) of individuals from households in the poorest wealth quintile experienced four months or more of insufficient food from subsistence agriculture. In terms of agricultural production, individuals from households that grew beans or maize were less likely to experience four months or more of insufficient food from subsistence agriculture compared to individuals from households that did not cultivate these staple crops. Additionally, 66.5% (95% CI: [63.1, 69.4]) of individuals from households without a migrant member experienced four months or more of insufficient food from subsistence agriculture, compared to 47.4% (95% CI: [41.6, 53.2]) of individuals from households with a migrant member.

Table 2. shows the results of the multivariate mixed effects negative binomial regression model and the associations between months with insufficient food availability and access from subsistence agriculture and demographic, socioeconomic, and agricultural production predictors. In line with the bivariate analysis, the prevalence of insufficient food availability and access was higher in larger and poorer households. Individuals from households with six to seven members had a 25% (PR = 1.25; 95% CI: [1.02, 1.54]) higher prevalence of insufficient food availability and access compared to individuals from households with one to three members. Individuals from households in the middle wealth quintile had a 20% (PR = 0.80; 95% CI: [0.65, 0.99]) lower prevalence of insufficient food availability and access compared to individuals from households in the poorest wealth quintile. Additionally, individuals from households that reported current problems with money had a 57% (PR = 1.57; 95% CI; [1.33, 1.85]) higher prevalence of insufficient food availability and access compared to individuals from households that did not report current problems with money.

Diversification of staple crops was associated with a significant reduction in the prevalence of insufficient food availability and access over the year. Individuals from households that grew both beans and maize had a 17% (PR = 0.83; 95% CI; [0.69, 0.99]) lower prevalence of insufficient food availability and access compared to individuals from households that did not grow these staple crops.

The prevalence of insufficient food availability and access was 20% (PR = 0.80; 95% CI: [0.70, 0.93]) lower among individuals from households that generated an income from agriculture compared to individuals from households that did not generate any income from agriculture. Conversely, individuals from households that generated income from local agricultural day labour had a 19% (PR = 1.19; 95% CI: [1.04, 1.36]) higher prevalence of insufficient food availability and access compared to individuals from households that did not generate income from this source. For individuals from households with a migrant member, there was no difference in the prevalence of seasonal food availability and access between individuals from households that received remittances from migrant labour and individuals from households that did not receive remittances. 

An examination of the random part of our multilevel negative regression models shows that the estimated variance for the random effect was significant, denoting heterogeneity in the prevalence of seasonal food availability and access at the household and community levels. Overall, 21% (σ^2^ = 0.21; 95% CI: [0.16, 0.27]) of the variance in observed seasonal food availability and access was at the household level and 3.8% (σ^2^ = 0.038; 95% CI: [0.014, 0.101]) of the variance in observed seasonal food availability and access was at the community level. 

## 4. Discussion

### 4.1. Climatic Conditions and Seasonality of Food Insecurity

A previous study in this region documented that the ‘lean’ season typically began in June and could last until September (four months) [12]. Our study found that approximately one quarter of households experienced insufficient food availability and access from subsistence agriculture in April and over half of households experienced insufficient food availability and access from subsistence agriculture in May. Moreover, approximately one quarter of households experienced insufficient food availability and access from subsistence agriculture for a six-month period (August–September 2013; April–July 2014).This potential lengthening of the ‘lean’ season is particularly problematic for food security, as seasonal food insecurity has been connected to decreasing caloric intake and worsening nutritional outcomes [7]. Seasonal hunger also negatively influences long-term health, labour productivity, and household finances, which in turn could exacerbate food insecurity [7,35]. In the context of longer and more severe drought in Honduras, it is important to consider the impacts of climatic changes on the seasonality of food insecurity, and how these changes may impact the way food insecurity is understood and experienced by subsistence agricultural producers.

The period during which this study was conducted marked the beginning of one of the most severe droughts in Honduras’ history [36], which created difficult agricultural conditions for small-scale subsistence farmers [9]. In 2013–14, lower than typical rainfall throughout Honduras coincided with planting and harvesting periods for maize and bean production, resulting in widespread crop losses and rising prices of staple foods [27,28,37]. Such conditions negatively impacted food availability and accessibility for households reliant on small-scale subsistence agriculture [37]. 

### 4.2. Factors Associated with Seasonal Food Insecurity

Beyond the climatic factors associated with food stability for small-scale subsistence farmers in rural areas of the Global South, there are a number of demographic, socioeconomic, and agricultural production factors influencing seasonal food availability, accessibility, and utilization [38,39]. In this study, individuals from larger and poorer households reliant on subsistence agriculture were more likely than individuals from smaller and wealthier households to have insufficient food availability and access. This is consistent with other studies in Latin America, in which overcrowding and low socioeconomic status have been cited as factors contributing to household food insecurity [14,40,41]. Beyond the total number of people in the household, household life cycle factors including household composition, age of household members, and marital status also influence individual and household food security [41,42,43]. Additionally, as gender influences food production, distribution, and consumption, it also has direct implications for individual and household food security [4]. In Latin America women are, in general, more likely to experience food insecurity than men, with this trend being more prominent in rural regions [41,43]. Although this study did not identify differences in seasonal food availability and access between women and men, further research is needed to more closely examine the relationship between the intrahoushold life cycle, gender, and seasonal food insecurity in this context.

The type of crops produced was also an important factor in understanding seasonal food availability and access. Specifically, our findings showed that individuals from households that grew maize and beans had lower levels of seasonal food insecurity. In rural Honduras, maize holds significance beyond its nutritional value, with maize playing an important cultural role in communities. For example, a study in rural Honduras found that hunger was often described by participants in terms of having limited access to maize (or tortillas made with maize flour), rather than the complete absence of food [2]. Understanding the cultural value of specific crops together with the amount of a crop being cultivated is important to consider when examining seasonal food insecurity for households reliant on small-scale subsistence agriculture. 

Livelihood diversification was further identified as an important factor influencing seasonal food insecurity. Individuals from households that generated income from subsistence agriculture were more food secure than households that did not. Generating income from subsistence agriculture demonstrated that these individuals were not only producing enough food to meet their families’ needs, but also generating a surplus of crops which enabled them to sell their produce. As such, the ability of subsistence agricultural producers to generate income from agriculture can be seen as an indication of household wealth and productivity. Conversely, households that used day labour as a livelihood diversification strategy had a higher prevalence of food insecurity. Although participation in day labour is an important form of livelihood diversification which can be used to meet food security needs [13], it may also be an indication that a household does not have sufficient land, or that their land is not productive enough to support surplus labour within a household.

Consistent with the results of a study on seasonal food insecurity in western El Salvador [1], individuals from households with a migrant member, in addition to individuals from households that received remittances from a migrant member, did not experience a significant increase in seasonal food availability and access. This finding may indicate that remittances are insufficient to influence subsistence agricultural production, that remittances were not being invested into subsistence agriculture, or that migration diverts labour away from subsistence agriculture resulting in fewer agricultural labourers for a household.

### 4.3. Strategies that Households Use for Food Availability and Food Access 

**Figure 2:**
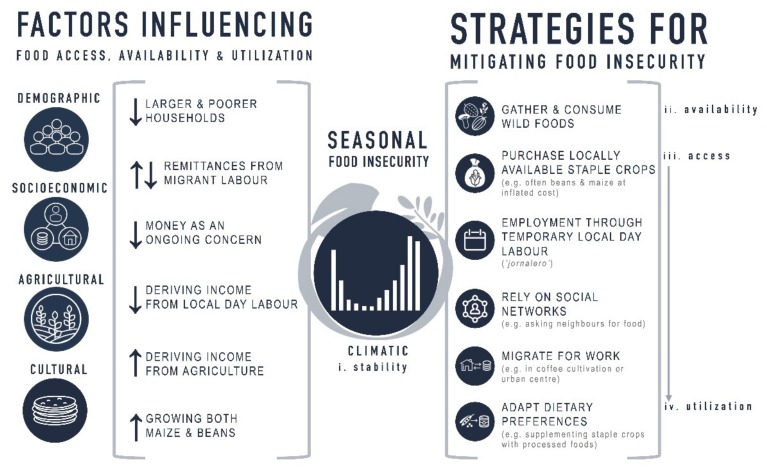
Factors influencing seasonal food insecurity among individuals from small-scale subsistence farming households in Yorito, Honduras. ↓ indicates a factor is associated with a decreased prevalence of sufficient food availability and access. ↑ indicates a factor is associated with an increased prevalence of sufficient food availability and access. ↓↑ indicates a factor is not significantly associated with the prevalence of sufficient food availability and access.

During times when subsistence agricultural production does not meet household needs, households are faced with the challenge of prioritizing resources between producing crops for income or for household food security [1]. Globally, households involved in subsistence agriculture have used a number of strategies to cope with seasonal food insecurity, such as: crop storage, selling livestock, selling assets, borrowing money and food, careful household administration, savings, farm diversification, off-farm income, changing diet, and early harvesting [1,6,7,44]. In the region where this study was conducted, there are several strategies employed by these households to mitigate seasonal food insecurity. To address food availability, household members may gather and consume wild foods (e.g., mushrooms, roots). To address food access, households may purchase locally available staple crops (beans and maize), often at inflated costs. Alternatively, households may seek employment through temporary local day labour (*jornalero*), rely on social networks (e.g., asking neighbours for food), or send one or several household members to migrate for work in either coffee cultivation or to an urban centre. Another potential strategy to enhance household food security was identified as adapting dietary preferences, such as supplementing staple crops with processed food (e.g., enriched white pasta, enriched white bread, fried snacks). Notably, seasonal food insecurity in rural areas of Honduras may contribute, in part, to the nutritional transition to more processed and often lower-quality foods that is already underway [45,46]. Despite potential changes to dietary preferences, this study found that households involved in small-scale subsistence agriculture considered themselves as food secure when they produced sufficient subsistence crops to meet their household consumption needs. In particular, an important component of food security was consuming culturally significant staple foods such as beans and maize tortillas. As such, while the strategies mentioned above may positively contribute to increasing food availability and access, they may also fall short of enabling households reliant on subsistence agriculture to utilize food in a preferred and culturally significant way (see Figure 2 for relevant factors that individuals and households need to navigate seasonal food insecurity). 

### 4.4. Limitations

This study has several limitations to consider when interpreting its findings. First, one member of each household was surveyed and acted as the proxy respondent for all members in their household. Information on household seasonal food availability and access provided by this individual was then extrapolated to all members of the household. Using this approach, we were able to investigate how the general life cycle and gender dynamics within households were associated with seasonal food availability and access. However, because we did not ask each individual member about food availability and access, we were unable to thoroughly examine the intrahousehold allocation of food, and account for situations where one household member received less or more food than other members. Despite this limitation, other tools that assess food security status (e.g., the United States Department of Agriculture Household Food Security Screener) use a proxy respondent approach in order to reduce the burden associated with survey administration on participating households. Second, as a result of collinearity among variables related to land ownership and crop type, we were unable to assess the association between the amount of land a household owned and seasonal food availability and access in the multivariable modeling. Additionally, we did not collect data on the diverse land tenure arrangements for small-scale subsistence farmers in this setting. Based on the bivariate analysis and other similar studies from the Global South (e.g., [38]), we expect that both the amount of land that households have access to, and the household’s land tenure arrangement, would influence seasonal food access and availability. Third, we did not examine the relationship between local meteorological data and seasonal food availability and access. This information would be helpful to explore if self-reported seasonal food availability and access is correlated with fluctuations in localized weather patterns. Finally, the findings from this study are specific to the region studied and cannot be extrapolated to all areas of rural Honduras. Notably, rural communities in the ‘dry corridor’ in Western Honduras have experienced successive droughts over the past decade, seriously compromising individual and household food security [47]. This reality underscores the importance of understanding within- as well as cross-country regional variation in order to inform agricultural and community development efforts and policies.

## 5. Conclusions

Our study contributes to an enhanced understanding of seasonal food insecurity in Central America. Of note, while seasonal food insecurity among small-scale farming households has previously been documented in Honduras and elsewhere in Central America, our study suggests that there may be a lengthening of or increased variability in the ‘lean’ season. Further research is needed to monitor local meteorological data to better understand how seasonal food insecurity is influenced by the interaction between climatic factors and the demographic, socioeconomic, and agricultural production factors examined in this study. Additionally, more research is needed to better understand how the life cycle of a household together with intrahousehold gender dynamics may influence experiences of seasonal food insecurity in this setting.

Integrating the analysis of seasonal food availability and access among small-scale subsistence farmers with an explicit consideration of the cultural value and use of staple subsistence crops allows us to understand the demographic, socioeconomic, and agricultural production factors that are associated with seasonal food insecurity in this setting. These factors modify the ability of individuals and households to navigate periods of seasonal food insecurity, and also influence their capacity to use the different strategies identified through this study to mitigate seasonal food insecurity.

In the context of a changing climate, nutritional transition, and the increased pressure toward internal and international migration from Central America, our findings underscore the need for complex policies and interventions that consider the interplay of climatic, cultural, socioeconomic, demographic, and agricultural production factors to address seasonal food insecurity. Additionally, our findings highlight the importance of accounting for food appropriateness and preference when seeking to understand the seasonal food security needs of small-scale subsistence farming households. These policies and interventions must be informed by the insights and experiences of small-scale subsistence farming households, and create space for their meaningful participation to share how they navigate the changing landscape of seasonal food insecurity. 

## Figures and Tables

**Table 1:** Bivariate associations between demographic, socioeconomic, and agricultural production factors and the number of months with insufficient food availability and access from subsistence agriculture (August 2013–July 2014) in Yorito, Honduras.

Characteristics	No Month Without Food	1–3 Months Without Food	4 Months and More Without Food	*P-Value*
	Percent (95% confidence interval, CI)	Percent (95% CI)	Percent (95% CI)	
Overall	4.43 [3.43, 5.72]	33.33 [30.78, 35.99]	62.23 [59.52, 64.87]	
Sex				0.979
Female (n = 624)	4.33 [2.98, 6.24]	33.33 [29.74, 37.14]	62.34 [58.46, 66.07]	
Male (n = 637)	4.55 [3.18, 6.48]	33.44 [29.87, 37.20]	62.01 [58.17, 65.71]	
Household size				<0.001
1 to 3 members (n = 144)	3.47 [1.45, 8.10]	42.36 [34.53, 50.60]	54.17 [45.95, 62.16]	
4 to 5 members (n = 486)	4.53 [3.00, 6.78]	33.54 [29.47, 37.87]	61.93 [57.52, 66.16]	
6 to 7 members (n = 372)	5.11 [3.28, 7.88]	24.73 [20.60, 29.38]	70.16 [65.30, 74.60]	
8 members & more (n = 261)	3.83 [2.07, 6.98]	40.23 [34.43, 46.31]	55.94 [49.84, 61.86]	
Marital status				<0.001
Single (n = 115)	6.09 [2.92, 12.26]	53.04 [43.87, 62.01]	40.87 [32.23, 50.11]	
Married (n = 708)	6.25 [3.09, 6.16]	31.5 [28.17, 35.02]	64.12 [60.51, 67.58]	
Widow/Widower (n = 15)	26.67 [10.00, 54.34]	20.00 [6.31, 48.12]	53.33 [28.55, 76.58]	
Divorced/Separated (n = 352)	3.41 [1.94, 5.91]	31.82 [27.15, 36.88]	64.77 [59.62, 69.60]	
Literacy				0.062
Can read and write (n = 237)	2.11 [0.88, 4.98]	31.65 [26.02, 37.86]	66.24 [59.97, 72.00]	
Illiterate (n = 127)	7.09 [3.72, 13.10]	39.37 [31.23, 48.15]	53.54 [44.81, 62.07]	
Can read, cannot write (n = 841)	4.76 [3.51, 6.42]	32.82 [29.72, 36.07]	62.43 [59.09, 65.64]	
Wealth index				<0.001
Poorest (n = 226)	0.00	27.43 [22.00, 33.64]	72.57 [66.36, 78.00]	
Poorer (n = 240)	0.00	36.67 [30.79, 42.97]	63.33 [57.03, 69.21]	
Middle (n = 262)	5.34 [3.19, 8.83]	35.88 [30.28, 41.89]	58.78 [52.70, 64.60]	
Richer (n = 264)	7.58 [4.93, 11.46]	28.79 [23.63, 34.56]	63.64 [57.64, 69.23]	
Richest (n = 271)	8.12 [5.40, 12.03]	37.27 [31.70, 43.20]	54.61 [48.63, 60.46]	
Current money problems				<0.001
No (n = 303)	16.50 [12.72, 21.13]	41.58 [36.15, 47.23]	41.91 [36.47, 47.56]	
Yes (n = 960)	0.63 [0.28, 1.39]	30.73 [27.89, 33.73]	68.65 [65.63, 71.51]	
Current land problem				<0.001
No (n = 840)	5.95 [4.54, 7.77]	38.10 [34.86, 41.43]	55.95 [52.57, 59.28]	
Yes (n = 423)	1.42 [0.64, 3.13]	23.88 [20.05, 28.18]	74.70 [70.33, 78.63]	
Current food problem				<0.001
No (n = 596)	8.39 [6.41, 10.91]	41.28 [37.38, 45.28]	50.34 [46.32, 54.35]	
Yes (n = 667)	0.90 [0.40, 1.99]	26.24 [23.03, 29.72]	72.86 [69.35, 76.11]	
Source of income is agriculture				<0.001
No (n = 544)	3.68 [2.38, 5.63]	26.10 [22.58, 29.97]	70.22 [66.23, 73.92]	
Yes (n = 719)	5.01 [3.63, 6.87]	38.80 [35.30, 42.43]	56.19 [52.53, 59.78]	
Income generation in the last year from local labour				<0.001
No (n = 538)	8.74 [6.62, 11.44]	41.45 [37.35, 45.67]	49.81 [45.59, 54.04]	
Yes (n = 725)	1.24 [0.65, 2.37]	27.31 [24.18, 30.68]	71.45 [68.04, 74.62]	
Receive remittances from migrant member				<0.001
No (n = 1,029)	2.82 [1.96, 4.03]	29.93 [27.21, 32.81]	67.25 [64.32, 70.05]	
Yes (n = 234)	11.54 [8.02, 16.32]	48.29 [41.93, 54.71]	40.17 [34.06, 46.60]	
Household has a migrant member				<0.001
No (n = 980)	2.96 [2.06, 4.23]	30.51 [27.70, 33.47]	66.53 [63.51, 69.42]	
Yes (n = 283)	9.54 [6.62, 13.57]	43.11 [37.44, 48.96]	47.35 [41.58, 53.19]	
Land ownership				<0.001
Not own land (n = 363)	3.58 [2.09, 6.08]	23.42 [19.33, 28.06]	73.00 [68.19, 77.33]	
Own land (n = 900)	4.78 [3.56, 6.38]	37.33 [34.23, 40.55]	57.89 [54.63, 61.08]	
Amount of land under cultivation (manzanas)				<0.001
No land (n = 380)	3.42 [1.99, 5.81]	23.16 [19.18, 27.68]	73.42 [68.74, 77.63]	
0.25–2 manzanas (n = 660)	2.73 [1.72, 4.29]	41.36 [37.66, 45.17]	55.91 [52.09, 59.66]	
Greater than 2 manzanas (n = 223)	11.21 [7.68, 16.08]	26.91 [21.48, 33.13]	61.88 [55.32, 68.04]	
Grow beans				<0.001
No (n = 892)	2.47 [1.63, 3.72]	32.29 [29.29, 35.43]	65.25 [62.05, 68.31]	
Yes (n = 371)	9.16 [6.62, 12.56]	35.85 [31.12, 40.87]	54.99 [49.88, 59.99]	
Grow maize				<0.001
No (n = 702)	1.85 [1.08, 3.17]	22.93 [19.97, 26.20]	75.21 [71.88, 78.27]	
Yes (n = 561)	7.66 [5.73, 10.18]	46.35 [42.25, 50.50]	45.99 [41.89, 50.14]	
Crop diversification				<0.001
Beans and maize not grown (n = 638)	23.21 [13.98, 36.00]	36.34 [31.88, 41.05]	60.05 [56.58, 63.42]	
Grow beans & maize (n = 307)	60.71 [47.47, 72.55]	29.69 [25.52, 34.24]	18.83 [16.24, 21.72]	
Grow beans or maize (n = 318)	16.07 [8.57, 28.11]	33.97 [29.59, 38.63]	21.12 [18.40, 24.12]	
Grow commercial (cash) crops				<0.001
No (n = 733)	6.00 [4.49, 7.97]	39.70 [36.21, 43.30]	54.30 [50.67, 57.88]	
Yes (n = 530)	2.26 [1.29, 3.95]	24.53 [21.05, 28.38]	73.21 [69.27, 76.81]	

**Table 2:** Results of the mixed effects negative binomial regression of the demographic, socioeconomic, and agricultural production determinants of insufficient food availability and access in Yorito, Honduras (August 2013–July 2014).

Characteristics	Crude Prevalence Ratio (PR) [95%CI]	Adjusted PR [95%CI]
Household size		
1 to 3 members	1	1
4 to 5 members	1.13 [0.95, 1.34]	1.21 [1.01, 1.46] *
6 to 7 members	1.14 [0.93, 1.39]	1.25 [1.02, 1.54] *
8 members and more	0.98 [0.76, 1.24]	1.07 [0.84, 1.36]
Wealth index		
Poorest	1	1
Poorer	0.99 [0.82, 1.20]	0.99 [0.81, 1.21]
Middle	0.79 [0.65, 0.95] *	0.80 [0.65, 0.99] *
Richer	0.70 [0.57, 0.86] ***	0.82 [0.67, 1.00]
Richest	0.78 [0.64, 0.94] *	0.9 [0.72, 1.11]
Current money problems		
No	1	1
Yes	1.58 [1.32, 1.90] ***	1.57 [1.33, 1.85] ***
Current land problem		
No	1	1
Yes	1.12 [0.96, 1.32]	1 [0.85, 1.17]
Crop diversification		
Beans & maize not grown	1	1
Grow beans and maize	0.71 [0.59, 0.85] ***	0.83 [0.69, 0.99] *
Grow beans or maize	0.83 [0.70, 0.99] *	0.89 [0.75, 1.05]
Source of income is agriculture		
No	1	1
Yes	0.77 [0.67, 0.89] ***	0.80 [0.70, 0.93] **
Income generation in the last year from local labour		
No	1	1
Yes	1.28 [1.12, 1.47] ***	1.19 [1.04, 1.36] *
Receiving remittances from migrant member		
No	1	1
Yes	0.88 [0.71, 1.08]	0.84 [0.70, 1.01]
Household has a migrant member		
No	1	
Yes	0.92 [0.75, 1.12]	
Current food problem		
No	1	
Yes	1.18 [1.00, 1.38] *	
Land ownership		
Not own land	1	
Own land	0.77 [0.67, 0.88] ***	
Amount of land under cultivation (manzanas)		
No manzana	1	
0.25–2 manzanas	0.82 [0.70, 0.96] *	
2.1 manzanas and more	0.60 [0.49, 0.73] ***	
Grow beans		
No	1	
Yes	0.79 [0.67, 0.93] **	
Grow maize		
No	1	
Yes	0.75 [0.65, 0.86] ***	
Grow commercial (cash) crops		
No	1	
Yes	0.91 [0.78, 1.07]	
Random Effects		
Community level		0.038 [0.014, 0.10]
Household level		0.21 [ 0.16, 0.27]

* *p* < 0.05; ** *p* < 0.01; *** *p* < 0.001.
